# Ketogenic diet: a potential adjunctive treatment for substance use disorders

**DOI:** 10.3389/fnut.2023.1191903

**Published:** 2023-07-27

**Authors:** Deshenyue Kong, Jia-xue Sun, Ji-qun Yang, Yuan-sen Li, Ke Bi, Zun-yue Zhang, Kun-hua Wang, Hua-you Luo, Mei Zhu, Yu Xu

**Affiliations:** ^1^General Hospital of Eastern Theater Command, Nanjing, China; ^2^Yunnan Technological Innovation Centre of Drug Addiction Medicine, Yunnan University, Kunming, China; ^3^First Affiliated Hospital of Kunming Medical University, Kunming, China; ^4^Third People’s Hospital of Kunming City/Drug Rehabilitation Hospital of Kunming City, Kunming, China

**Keywords:** ketogenic diet, substance use disorders, addiction, metabolism, neuroprotection, gut microbiota

## Abstract

Substance use disorders (SUD) can lead to serious health problems, and there is a great interest in developing new treatment methods to alleviate the impact of substance abuse. In recent years, the ketogenic diet (KD) has shown therapeutic benefits as a dietary therapy in a variety of neurological disorders. Recent studies suggest that KD can compensate for the glucose metabolism disorders caused by alcohol use disorder by increasing ketone metabolism, thereby reducing withdrawal symptoms and indicating the therapeutic potential of KD in SUD. Additionally, SUD often accompanies increased sugar intake, involving neural circuits and altered neuroplasticity similar to substance addiction, which may induce cross-sensitization and increased use of other abused substances. Reducing carbohydrate intake through KD may have a positive effect on this. Finally, SUD is often associated with mitochondrial damage, oxidative stress, inflammation, glia dysfunction, and gut microbial disorders, while KD may potentially reverse these abnormalities and serve a therapeutic role. Although there is much indirect evidence that KD has a positive effect on SUD, the small number of relevant studies and the fact that KD leads to side effects such as metabolic abnormalities, increased risk of malnutrition and gastrointestinal symptoms have led to the limitation of KD in the treatment of SUD. Here, we described the organismal disorders caused by SUD and the possible positive effects of KD, aiming to provide potential therapeutic directions for SUD.

## 1. Introduction

Substance use disorders (SUD) are chronically relapsing conditions characterized by compulsive substance-seeking, lack of control in restricting intake, and the development of negative emotions during withdrawal (1). According to 2021 National Survey on Drug Use and Health in the United States, 46.3 million people aged 12 or older (16.5%) had SUD in the past year, including 29.5 million people with alcohol use disorders (AUD) and 24 million people with drug use disorders (DUD), and 7.3 million people have both AUD and DUD (2). A study based on the Global Burden of Disease showed that the global prevalence of substance use disorders was approximately 2.2%, with the prevalence of AUD (1.5%) being higher than other DUD (0.8%, including cannabis 0.32%, opioids 0.29%, amphetamines 0.10%, and cocaine 0.06%) (3). SUD is a significant contributor to the global burden of disease (4, 5). Deaths from SUD are increasing, rising from approximately 284,000 deaths in 2007 to 352,000 in 2017, creating a global health threat (6). Commonly abused substances include alcohol, opioids, stimulants, etc., which cause damage to various systems of the body, mainly the nervous system (7) and the immune system (8), etc. In addition, they often lead to metabolic disorders (9, 10). Many treatments for SUD are available, including non-pharmacological treatments, such as cognitive-behavioral therapy, and pharmacological treatments, such as drug replacement therapy including methadone, buprenorphine, or naltrexone maintenance (11). However, a radical cure for SUD usually means abstaining from substance abuse and dependence, but relapse is common and a radical cure for such disorders has not yet been discovered. Similar to other chronic conditions like heart disease or asthma, the treatment goal for SUD is typically not a radical cure but rather managing it as a chronic condition through a combination of complementary methods (12). However, the efficacy and sufficiency of SUD treatment strategies are affected by a multitude of factors. Firstly, SUD entails the interaction of multiple factors encompassing physical, psychological, and social domains; thus, a comprehensive treatment approach must account for these multifaceted dimensions. Second, there is individual variability in SUD, requiring individualized treatment plans. Third, different substance use disorders, such as alcohol and drugs, have specific treatment strategies and require targeted programs. Fourth, some individuals may encounter barriers to accessing appropriate treatment, such as limited medical resources, thereby impeding their ability to receive effective care. Furthermore, there exist challenges pertaining to individuals with SUD themselves; for example, an estimated 94% of individuals with SUD aged 12 or older in the United States do not receive any form of treatment, and almost all those who do not receive treatment at a specialty facility believe they do not need treatment (2). SUD remains severely undertreated, and it is estimated that only 11% of substance use individuals in need of treatment received appropriate care in the United States (13). Treatment of SUD is a long-term process, and it may be more effective to consider SUD as a chronic disease and to treat them in combination with multiple supporting approaches.

The ketogenic diet (KD) is a diet with a reduced proportion of carbohydrates and a relatively increased proportion of fat (14). KD causes a fasting-like effect and puts the body into a state of ketosis. Normally, carbohydrates are converted to glucose as the main source of energy for the brain. However, in the absence of carbohydrates, the body begins to look for alternative sources of energy (15), namely from acetyl coenzyme A producing excess ketone bodies, including acetoacetate, β-hydroxybutyric acid (BHBA), and acetone. This process is called ketogenesis (16). Unlike pathological keto acidosis, ketosis is a physiological mechanism, as these ketone bodies can be efficiently used without excessive concentrations (17). In the past, KD has been widely and successfully used in the treatment of epileptic disorders and obesity (18, 19). In addition, there has been increasing evidence in recent years of the therapeutic potential of KD in other diseases, such as diabetes (20), Alzheimer’s disease (21), Parkinson’s disease (22), cancer (23), autism (24), multiple sclerosis (25), and headache (26). Most of them were neurological disorders, suggesting a potential role for the ketogenic diet in these disorders (27). Recent research has found that KD may also have a positive effect on SUD, mainly in the treatment of AUD (28). In addition, cocaine-related animal studies have shown that KD decreased cocaine-induced stereotyped responses in rats, and also disrupted the sensitization of ambulatory responses (29). Here, we summarize the current studies on the treatment of SUD by KD (Table 1). These have led to a further understanding of the therapeutic potential of KD.

**Table 1:** Clinical and preclinical studies reporting the effect of the KD on SUD.

Substance	Clinical/preclinical	Methods of KD	Major outcome	References
	Clinical study			
Alcohol	Patients with AUD (Standard American diet, *n* = 14; KD, *n* = 19)	80% fat, 15% protein, and 5% carbohydrates; 3 weeks	Reduced alcohol craving and withdrawal symptoms (needed less benzodiazepines)	(28)
	Preclinical study			
Alcohol	Male OF1 mice (alcohol oral self-administration paradigm)	90.5% kcal from fat, 0.3% kcal from carbohydrates and 9.1% kcal from protein; 6.7 kcal/g; 4 weeks	Decreased oral ethanol self-administration	(30)
Alcohol	Male Wistar rats (alcohol self-administration)	5% of calories was from protein, 2% was from carbohydrates, and 93% was from fat; 8 weeks	Reduced alcohol consumption	(28)
Alcohol	Male Sprague Dawley rats (alcohol oral gavage for 6 days)	93% calories from fat, 5% from protein, 2% from carbohydrate; 10 days	Decreased alcohol withdrawal symptoms (“rigidity” and “irritability”)	(31)
Alcohol	Male C57BL/6NTac mice (intermittent alcohol exposure for 3 weeks using liquid diet)	93% calories from fat, 5% from protein, 2% from carbohydrate; 3 weeks	Decreased alcohol withdrawal symptoms (convulsions and anxiety-like behavior)	(32)
Cocaine	Sprague–Dawley rats (daily intraperitoneal injections of 15 mg/kg cocaine for 1 week, were drug free for a subsequent week, and then received a final challenge injection of 15 mg/kg cocaine)	93% kcal from fat, 2% kcal from carbohydrates and 5% kcal from protein; 5 weeks	Decreased cocaine-induced stereotyped responses; disrupted sensitization of ambulatory responses	(29)
Cocaine	Male OF1 mice (intraperitoneal injections of 10 mg/kg cocaine induced CPP procedure)	90.5% kcal from fat, 0.3% kcal from carbohydrates and 9.1% kcal from protein; 8 or 10 weeks	Reduced the number of sessions required to extinguish the drug-associated memories and blocked the priming-induced reinstatement.	(33)
Cocaine	Male C57BL/6J (intraperitoneal injections of 10 mg/kg cocaine induced CPP procedure; after extinction for 3 weeks, the mice received a priming dose of 7.5 mg/kg cocaine and underwent a reinstatement test)	90.5% kcal from fat, 0.3% kcal from carbohydrates and 9.1% kcal from protein; 3 weeks	Disrupted cocaine CPP reinstatement	(34)
Oxycodone	C57BL/6J mice (oxycodone self-administration)	93.4% fat, 4.7% protein, and 1.8% carbs; 4–14 days	Increased oxycodone-induced locomotor activity and enhanced antinociceptive effects of oxycodone; decreased oxycodone self-administration in male mice	(35)

KD, Ketogenic diet; SUD, substance use disorders; AUD, alcohol use disorders.

**Figure 1:**
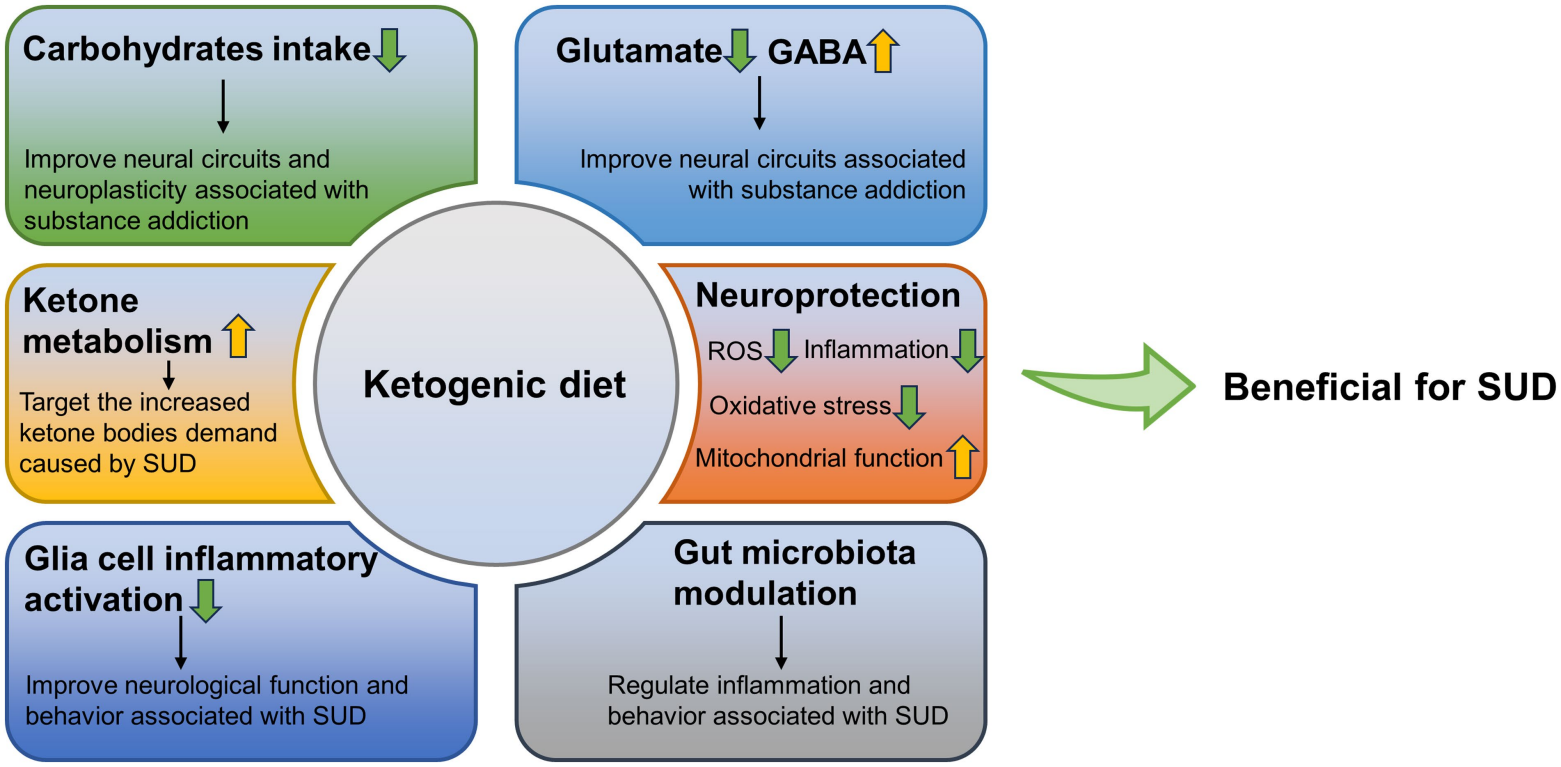
Possible positive effects of ketogenic diet against the negative effects caused by SUD. GABA, γ-aminobutyric acid; SUD, substance use disorders; ROS, reactive oxygen species.

KD may be effective as a potential adjunctive therapy for SUD treatment. Importantly, it may have targeted protective effects. Here, we summarized the possible positive effects of KD in reducing carbohydrate intake, ketosis status, neuroprotection, protecting glial cells, reducing inflammation, and regulating gut microbiota, against the negative effects caused by SUD (Figure 1). However, there are few relevant studies besides AUD, and the role of KD in the treatment of SUD has not been clarified. Although SUD treatments exist, current treatment strategies are still not effective and sufficient, and there is a clear need for more effective treatments (13). The purpose of this review is to evaluate the viability of KD as a potential therapeutic option, to provide potential directions for future treatment of SUD, and to alleviate the suffering caused by SUDs to individuals, as well as the huge toll they take on a social level.

## 2. Ketogenic diet decreases the intake of carbohydrates

Since the nutritional status of the SUD population usually appears abnormal (36), some studies have evaluated the dietary patterns of this population. The results show that SUD, particularly opioid use disorder, such as heroin use, and treatment with methadone, are strongly associated with increased consumption of sugar or sweets (37–40). These people preferred sucrose-rich foods to protein-and fat-rich foods (37). Similarly, cocaine and alcohol abusers also preferred higher concentrations of sucrose and highly sweet foods (41–43). And after withdrawal, the dietary patterns improved (38). It has been shown that excessive intake of carbohydrates, whether monosaccharides (glucose and fructose), disaccharides (sucrose), or polysaccharides (starch and glycogen), can injure human health (44). Sugar intake is also associated with increased vulnerability to SUD. By analyzing data from 17 countries worldwide, sugar and sweetener supply quantity were significantly and positively associated with anxiety disorders, mood disorders, impulse control disorders, and SUD (45). Patients undergoing bariatric or weight loss surgery have a higher incidence of substance use disorders, which may be attributed to the intake of high-sugar/low-fat foods or foods with a high glycemic index (46). In addition, sweet liking status predicts an increased risk of having alcohol-related problems in young adults (47). Likewise, there is similar evidence in animal experiments. For example, high saccharin intake rats are a well-recognized model of vulnerability to drug abuse. High saccharin rats are more likely to consume sweets and cocaine and are more vulnerable to the negative emotional effects of morphine withdrawal (48, 49). Excessive sucrose intake disrupts reward processing and reduces the reward value of sucrose in rats (50). Mice fed a 10% sucrose solution for 4 weeks showed greater locomotor responses to acute cocaine use compared to mice consuming standard food and water (51). Therefore, this particular dietary preference should receive attention, and exploring the reasons for increased sugar consumption and the mechanisms underlying the association between sugar intake and SUD may provide potential information to improve health.

The sweet taste preference of the SUD population makes it tempting to wonder whether their increased preference and consumption of sugar are associated with changes in gustatory responses. However, several studies on taste perception in SUD populations have shown different results, making it unclear whether SUD causes changes in taste perception that give rise to a preference process for sugar (52–54). These differential results may be attributed to the small sample size of the experiment, as well as differences in gender, age, education level, and income of participants. In addition, there are methodological differences between studies in terms of sucrose pleasantness and intensity measurements.

Importantly, the neural circuits associated with sugar intake may be similar to SUD. The mu-opioid receptor (MOR) is a G protein-coupled receptor that is associated with pain perception. It is not only a major target for heroin or other opioids, but also for most non-opioid substances of abuse, such as alcohol, cocaine, and nicotine (55, 56). Interestingly, several studies suggest a link between sugar or sweet foods intake and MOR. In rodent models, MOR knockout or antagonism leads to reduced hedonic responses to sweet stimuli and consumption of sweet solutions or sucrose (57–59). Similarly, in humans, morphine stimulation of MOR increased the pleasure of the sucrose solutions (60). In contrast, a MOR inverse agonist, GSK1521498, selectively reduced sensory hedonic ratings and intake of high-sugar foods (61). MOR in the nucleus accumbens (NAc) may partially mediate the motivation and hedonic experience of sucrose consumption (62). Specifically, the rostrodorsal quadrant of the NAc contains an opioid hedonic hotspot that mediates the enhancements of sucrose “liking” (63). Also, this is important for the endocannabinoid-enhanced preference for sucrose (64). Suggesting the interdependence of opioid and cannabinoid signaling in enhancing taste hedonic.

Another important neural circuit is the dopaminergic system. The dopaminergic system is a key regulatory component of behavior associated with SUD. Almost all known addictive substances can cause a significant increase in dopamine (DA) release and activate reward areas in the brain (65, 66). The effects of substances of abuse include direct activation of DA neurons, increased DA release, blockade of DA reuptake, and DA neuron de-inhibition (67). Excessive DA signaling may regulate gene expression and modify the synaptic function and circuit activity (68). In addition, the substance of abuse act on the nucleus accumbens DA signaling, inducing glutamatergic-mediated neural adaptations in the DA striatal-thalamo-cortical and limbic pathways (69). These effects eventually lead to substance addiction. It has been shown that intermittent sucrose use and drug abuse are similar in that both can repeatedly increase extracellular DA in the NAc shell (70). And this sucrose-induced DA release in the NAc may be sucrose concentration-dependent (71).

Sucrose consumption may lead to pathophysiological consequences similar to those produced by substance abuse (72). High-fat and high-sugar diet alters glutamate, DA, and opioid signaling in the dorsal striatum of mice. Specifically, a high-fat and high-sugar diet increased the α-amino-3-hydroxy-5-methyl-4-isoxazole-propionic acid – to – N-methyl-D-aspartic acid receptor current ratio in medium spiny neurons and prolonged spontaneous glutamate-mediated currents, increased DA release and slower DA reuptake in the striatum, and reduced MOR-mediated synaptic plasticity (73). In this study, both high fat and high sugar were added to the diet, and it is not clear which of these factors played a major role. However, Study shows that sucrose intake reduces the availability of MOR and DA D2/3 receptors in the porcine brain (74). In addition, in rats, casual exposure to 1% sucrose for 3 weeks resulted in altered MOR and D1/D2 receptor mRNA and protein expression in the NAc (75). And repeated sucrose intake decreased the density of DA D2 receptors in the striatum (76). Thus, sucrose may alter the neural circuits that encode reward and enhance the SUD drive.

**Figure 2:**
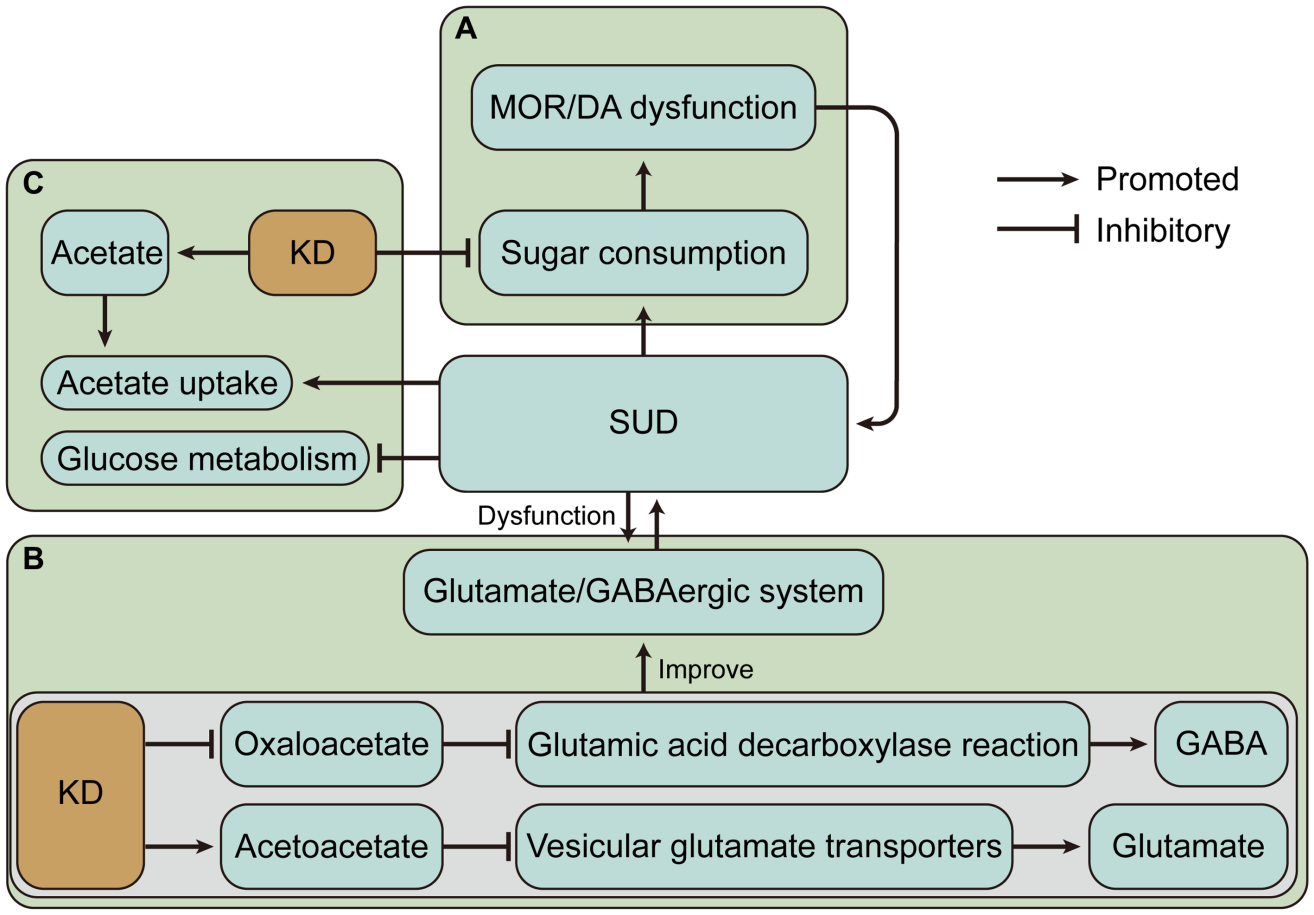
The ketogenic diet (KD) may have beneficial effects on substance use disorders (SUD) by altering dietary intake and changing metabolic status. **(A)** KD may alleviate the vicious cycle caused by SUD and sugar intake. **(B)** KD may alleviate SUD-induced glutamate/GABAergic disorders by decreasing glutamate and increasing GABA. **(C)** Ketone bodies produced by KD may alleviate impaired glucose metabolism and increased ketone body requirements due to SUD. MOR, mu-opioid receptor; DA, dopamine; GABA, γ-aminobutyric acid.

The SUD and increased sugar intake appear to be a vicious cycle due to similar neural circuits and altered neuroplasticity. Sensitivity to one substance may lead to cross-sensitization to another substance. For instance, amphetamine treatment produced psychomotor sensitization and accelerated the subsequent escalation of cocaine intake (77), and nicotine-sensitive animals may consume more alcohol (78). Hence, increased sugar intake may also cause sensitivity to SUD. Reducing sugar intake may be an important complement to the treatment of SUD. And the very low carbohydrate intake of the ketogenic diet may offer help for this potential treatment (Figure 2A). However, the role of the KD in SUD treatment by lowering carbohydrate intake is largely unknown. Conversely, for example, fat consumption was also associated with the DA and opioid signaling pathways in the hypothalamus (79). Therefore, the improved effect of KD with low-carbohydrate and high-fat intake in the neural circuits of addiction remains to be investigated.

## 3. Ketogenic diet and glutamate/GABAergic system

DA is critical for acute reward and the onset of addiction, but the later stages of addiction are primarily related to glutamatergic projections. Dysregulation of glutamate, an excitatory neurotransmitter, leads to changes in neuroplasticity, which reduces the value of natural rewards, decreases cognitive control, and promotes compulsive drug-seeking (80–82). Imbalance in glutamate homeostasis is a key neurometabolic feature of SUD. By a proton magnetic resonance spectroscopy protocol, it was found that basal glutamate concentrations in the NAc were significantly lower in cocaine addicts and that glutamate levels increased during cue-induced cravings in cocaine-addicted individuals compared to baseline (83). Changes in relapse behavior induced by activation or inhibition of glutamate receptors suggest that glutamate may be the major mediator of substance-seeking behavior recovery. For example, stimulation of group II metabotropic glutamate receptor and blockade of group I metabotropic glutamate receptor could prevent relapse of cocaine, heroin, nicotine, and alcohol (84–90).

The rapid elevation and release of DA in the voxel nucleus are important causes of the reward response in addiction which can be antagonized by increasing γ-aminobutyric acid (GABA) levels (91). Diminished GABA signaling leads to increased susceptibility to SUD. For example, the excitatory signal from the entopeduncular nucleus to the lateral habenula is limited by GABAergic cotransmission, but during cocaine withdrawal, the presynaptic vesicular GABA transporter is reduced, resulting in a decrease in this inhibitory component and causing relapse. And stress-induced relapse can be prevented by restoring GABAergic neurotransmission (92). GABA release in DA neurons is dependent on the GABA synthesis pathway mediated by aldehyde dehydrogenase 1a1 and decreased it leads to increased alcohol consumption and preference (93). In addition, exposure to morphine blocks the long-term potentiation of GABA-mediated synaptic transmission and may contribute to the development of addiction (94).

KD may alleviate SUD by decreasing glutamate and increasing GABA. A similar example is that KD significantly increased the level of GABA in cerebrospinal fluid in children with refractory epilepsy (95). Previous studies have shown that KD treatment increases GABA levels in the hippocampus of the rat brain (96). The possible mechanism is that KD may limit the availability of oxaloacetate in the aspartate aminotransferase reaction, allowing more glutamate to enter the glutamic acid decarboxylase reaction and produce GABA (97). KD also produces ketone body carbon which is metabolized to glutamine, the basic precursor of GABA, in addition to the increased consumption of acetate in ketosis, which is converted to glutamine via astrocytes in the brain (97). Relatively, the glutamate level in the brain of rats receiving KD was reduced (98). It has also been proposed that KD intervention for anxiety and depression in Alzheimer’s Disease may related to the modulation of the glutamatergic neurotransmission system (99). Acetone and BHB act as glutamate inhibitors in the N-methyl-D-aspartic acid receptor receptor (99, 100). Evidence suggests that acetoacetate inhibits glutamate release and another mechanism by which KD reduces glutamate is that Cl-activates vesicular glutamate transporters, while increased ketone bodies, especially acetoacetate, inhibit vesicular glutamate transporters by competing with Cl^−^, thereby inhibiting glutamate release (101) (Figure 2B). Overall, KD leads to an enhanced conversion of glutamate to glutamine, which permits a more efficient removal of glutamate and conversion of glutamine to GABA (102). These evidences provide rationale for the treatment of SUD by the glutamate/GABAergic system.

## 4. Ketogenic diet increases ketone metabolism

The KD creates a special metabolic state that changes the energy metabolism, which is dominated by glucose metabolism in the brain, to ketone body metabolism. Because of this particular state of energy metabolism that develops, the KD may be more effective in the treatment of AUD. The effects of alcoholism on brain glucose and acetate metabolism were assessed by positron emission tomography, and brain glucose metabolism was found to be reduced and acetate uptake was increased during alcoholism, suggesting that increasing acetate concentration through a KD may have therapeutic benefits for AUD (103). And several preclinical and clinical studies have provided a partial basis for this. KD decreased the rigidity and irritability symptoms and reduced convulsions and anxiety-like behaviors during the alcohol withdrawal (31, 32). And KD reduces alcohol consumption in alcohol-dependent rats (28). Mice exposed to a ketogenic diet exhibited an overall reduction in ethanol consumption during stable ketosis, while gene expression analysis revealed several changes in the DA, adenosine, and cannabinoid systems (30). In addition, ketogenic diet-induced fibroblast growth factor 21 administration significantly reduced sweet taste and alcohol preference in mice and sweet taste preference in cynomolgus monkeys, and was associated with reduced DA concentrations in the NAc (104). Human studies on AUD have shown that patients who received KD experienced decreased withdrawal symptoms, required less benzodiazepines, had reduced cravings for alcohol, and exhibited increased reactivity to alcohol cues in the dorsal anterior cingulate cortex (28). However, a case report showed that prolonged KD combined with alcohol intake can disrupt glucose homeostasis and lead to significant hypoglycemia (105), suggesting that counseling patients about alcohol intake during the KD is necessary.

During alcohol abuse, the demand for ketone bodies in the brain increases, and adaptation to repeated alcohol intake occurs, while acetate levels decline during withdrawal, and supplementation of ketone bodies through a KD may alleviate withdrawal symptoms due to the sudden shift in metabolic status (106). These may be the mechanisms by which the KD alleviates AUD. It is worth noting that several studies have shown that other substance use disorders also exist with impaired brain glucose metabolism. For example, regional cerebral glucose metabolism was lower in both opioid withdrawal and methadone maintenance subjects than in control subjects (107, 108). Cocaine and nicotine reduce brain glucose metabolism (109, 110). During methamphetamine abuse and withdrawal, glucose metabolism is reduced in several brain regions including the thalamus, striatum, and frontal lobes (111–113). The prevalence of impaired cerebral glucose metabolism suggests that KD may have beneficial effects by changing the major energy metabolism of the brain in SUD (Figure 2C).

## 5. Neuroprotective effects of the ketogenic diet

Mitochondria is an important organelle for energy production and is also involved in a series of processes of signaling and cell death. During oxidative metabolism and various cellular reactions, mitochondria produce reactive oxygen species (ROS), associated with the function of the mitochondrial electron transport chain. Due to the lack of adequate antioxidant systems, the brain and nervous system are easily affected by oxidative stress (114). Impaired mitochondrial redox homeostasis, such as oxidative stress due to excessive ROS production and impairment of antioxidant function, can cause mitochondrial dysfunction and the onset of the cell death cascade, leading to neuronal damage (115, 116). And oxidative stress can cause free radicals to attack nerve cells, leading to neurodegeneration (117). Substance abuse causes widespread neurotoxicity, mainly related to mitochondrial dysfunction and oxidative stress (118). In response to the dopaminergic neural activation induced by substances of abuse, such as heroin (119, 120), methamphetamine (121, 122), and cocaine (123), the mitochondrial respiratory chain is rapidly activated, leading to mitochondrial dysfunction, such as decreased mitochondrial membrane potential, mitochondrial DNA, and mitochondrial proteins, and increased cytochrome c release, resulting in increased ROS and oxidative stress (124).

**Figure 3:**
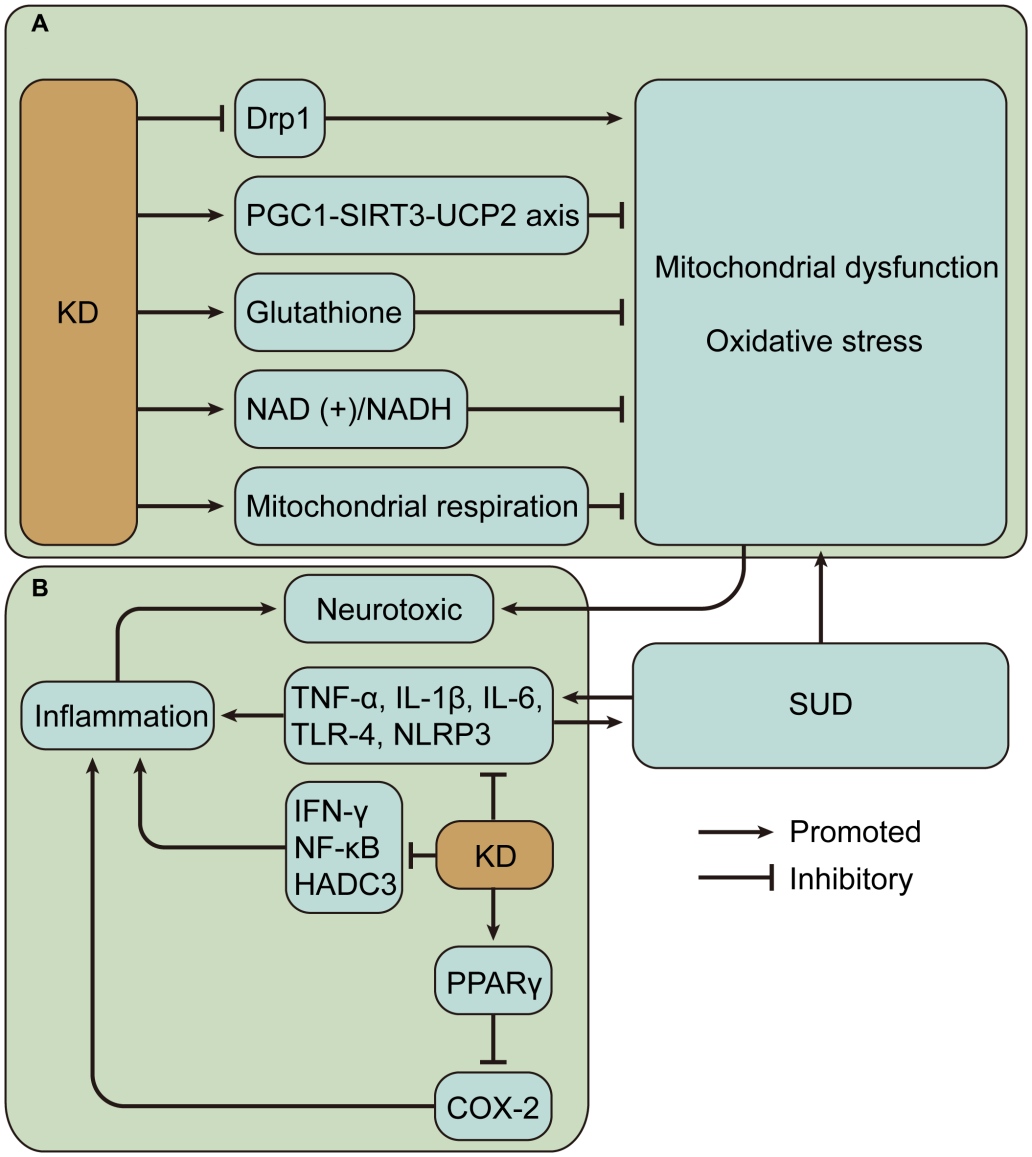
Potential mechanisms of neuroprotective effects of ketogenic diet (KD) in substance use disorders (SUD). **(A)** KD may alleviate mitochondrial dysfunction and oxidative stress caused by SUD. **(B)** KD may alleviate inflammation caused by SUD. Drp1, dynamin-related protein 1; PGC1-SIRT3-UCP2, peroxisome proliferator-activated receptor γ-coactivator-1α – sirtuin 3 – uncoupling proteins 2; NAD (+)/NADH, nicotinamide adenine dinucleotide (+)/nicotinamide adenine dinucleotide hydrogen; TNF, tumor necrosis factor; IL, interleukin; NLRP3, nucleotide-binding domain-like receptor protein 3; IFN, interferon; NF-κB, nuclear factor-kappa B; HADC3, histone deacetylase 3; PPAR γ, peroxisome proliferator-activated receptor γ; COX-2, cyclooxygenase-2.

KD is neuroprotective and, importantly, its primary activity may be associated with improved mitochondrial function and reduced oxidative stress (125). KD, especially BHBA, inhibits dynamin-related protein 1-mediated mitochondrial fission (126). Dynamin-related protein 1 is a GTPase that interacts with the mitochondrial fission protein Fis1 to induce mitochondrial fission, which produces ROS and activates nucleotide-binding domain-like receptor protein 3 (NLRP3) inflammasome (127, 128). Detection of mRNA and protein levels showed that KD or ketone bodies can improve mitochondrial biogenesis and bioenergetics through the peroxisome proliferator-activated receptor γ-coactivator-1α – sirtuin 3 – uncoupling proteins 2 axis, and uncoupling proteins upregulated by mitochondrial respiration can reduce the production of ROS and oxidative stress (129, 130). Similarly, enhanced mitochondrial biogenesis was verified by define of gene expression patterns in rat hippocampus at 3 weeks of KD (131). 3-week KD increased glutathione biosynthesis in rat hippocampal mitochondria, enhanced mitochondrial antioxidant status, and protected mitochondrial DNA from oxidant-induced damage (132). BHBA and acetoacetate reduce glutamate-induced free radical formation by increasing the nicotinamide adenine dinucleotide (+)/ nicotinamide adenine dinucleotide hydrogen ratio and strengthening mitochondrial respiration (133), and prevent mitochondrial permeability transition and oxidative injury (134, 135). In addition, KD reduces the release of mitochondrial cytochrome c, thus achieving a neuroprotective effect (136). These findings provide a wealth of evidence for a possible neuroprotective role of KD in SUD (Figure 3A).

Inflammation is another important factor in neurodegeneration, which may lead to the production of neurotoxic mediators (137). Many substances of abuse can lead to increased inflammation, mainly characterized by upregulation of pro-inflammatory cytokine levels, such as tumor necrosis factor (TNF)-α, Interleukin (IL)-1, and IL-6 (138–140). In addition, neuroinflammation in SUD can also be induced by the activation of toll-like receptor 4 receptors and NLRP3 inflammasome (141–143). Cytokines have been shown to influence host behavior (144). Inflammation appears to lead to persistent changes in basal ganglia and DA function, as shown by lack of pleasure, fatigue, and psychomotor slowing. And inflammation also leads to reduced neural responses to hedonic rewards, reduced DA metabolites, and increased reuptake and decreased turnover of presynaptic DA (145, 146). In SUD, inflammatory responses may contribute to drug-induced rewards and drug relapse. An interesting example is that cannabidiol may reduce METH relapse by improving cytokine expressions, such as IL-1β, IL-6, IL-10, and TNF-α (147).

Evidence suggests that KD reduces inflammation in a variety of diseases. KD activates peroxisome proliferator-activated receptor gamma to inhibit cyclooxygenase-2-dependent pathways and thereby suppress neuroinflammation in the mouse model of epilepsy (148). Inflammation is a key secondary pathological process in spinal cord injury, and KD attenuates inflammation, including reduced nuclear factor-kappa B pathway and expression levels of TNF-α, IL-1β, and interferon-γ (149). In colorectal tumor-bearing mice, the ketogenic formula suppressed systemic inflammation associated with tumors, such as lowering plasma IL-6 levels (150). In a demyelination mouse model with neuroinflammation, KD treatment inhibits the activation of pro-inflammatory glial cells and decreases the production of pro-inflammatory cytokines, including IL-1β and TNF-α, in addition to significantly reducing histone deacetylase 3 and NLRP3 (151). Histone deacetylases alter chromatin structure and accessibility, impairing memory function and synaptic plasticity (152). In a mouse model of Alzheimer’s disease, a 4-month KD reduced neuroinflammation, decreased microglia activation, and thus improved cognitive function (153). Single-cell RNA sequencing of adipose-resident immune cells showed that KD expanded metabolically protective γδ T cells and suppressed inflammation (154). The results suggest that KD mostly alleviates inflammation by reducing pro-inflammatory cytokine levels and plays a beneficial role in different diseases (Figure 3B). However, there are also contrary evidence suggesting the complexity of KD regulation of inflammation. One study evaluated postprandial responses in 17 men after transitioning from a baseline diet to an isocaloric KD and found increased markers of inflammation (155). Hyperketonemic diabetic patients have significantly higher levels of TNF-α and IL-6 than normoketonemic diabetic patients (156, 157). Short-term use of exogenous ketone supplements increases blood BHBA but leads to increased markers of NLRP3 inflammasome activation including caspase-1 and IL-1β secretion (158). In addition, it was found that high concentrations of BHB induced an increase in pro-inflammatory signals such as TNF-α, IL-6 and IL-1β (159). BHB can also mediate inflammation by promoting neutrophil adhesion through inhibition of autophagy (160). Therefore, it is crucial to measure ketosis levels, such as BHB levels, to properly evaluate the impact of KD.

According to the above results, KD may play an important protective role in SUD-mediated neurological damage mainly by protecting mitochondria and reducing oxidative stress and inflammation. However, different strategies, such as the timing of intervention, need to be considered during the KD.

## 6. Glial cells in the ketogenic diet

Glial cells account for about half of the central nerve cells, and they influence the formation and function of the neural system (161). Glial cells regulate neurotransmission, synaptic connections, and neural circuits, which can influence brain function and behavior in SUD (162–164). In the past, microglia were usually thought of as phagocytes in the central nervous system; however, in fact, recent studies have revealed that microglia are important participants in central nervous system homeostasis, and their dysregulation causes neurological diseases (165, 166). Microglia may regulate neuronal function and be associated with the development of SUD. Chronic high-dose alcohol intake induces reactive gliosis and may increase the risk of dementia (167). In contrast, depletion of microglia reduced alcohol consumption in mice, decreased anxiety-like behavior, and reduced GABA and glutamate receptor-mediated synaptic transmission in the central nucleus of the amygdala (168). Morphine withdrawal in mice leads to microglia adaptations and reduced glutamatergic transmission, resulting in reduced synaptic excitation and social behavior (169). Many substances of abuse cause neurological dysfunction by activating microglia. For example, cocaine may activate microglia by downregulating miR-124 targeting kruppel-like factor 4 and toll-like receptor 4 signaling, which may contribute to cocaine-induced synaptic plasticity (170, 171). Methamphetamine activates the NLRP3 inflammasome in microglia and promotes the processing and release of IL-1β, resulting in neurotoxicity (142). In addition to microglia, another type of glial cell in the central nervous system, astrocytes, is also important in neurological diseases (172, 173). Interestingly, MOR is highly expressed in astrocytes (174). Activation of astrocyte MOR elevates cytosolic calcium ions, leading to rapid glutamate release, and further regulating neuronal activity (175, 176). The activation of astrocytes may be involved in the addiction process. Studies have shown that glutamate released by astrocyte MOR activation enhances synaptic transmission and can drive conditioned place preference (CPP) (177). In addition, activation of astrocytes is associated with neuroinflammation (178, 179). For example, morphine activates astrocytes and promotes the production of pro-inflammatory cytokines (180). There are also interactions between glial cells. Morphine-mediated release of miR-138 from astrocyte-derived extracellular vesicles promotes microglia activation and further causes neurological dysfunction (181). Thus, glial cell disorders play a key role in SUD, and improving glial cell disorders may facilitate recovery from SUD.

In a study of normal adult rats, KD was able to alter glial morphology, suggesting that KD may have neuroprotective effects by affecting glial cells (182). In a variety of disease models, KD has played a similar role in reducing glial cell activation. Studies have shown that KD reduced the inflammatory activation of microglia in glaucoma, depression, and multiple sclerosis mouse models (183–185). Similarly, activation of reactive astrocytes can be inhibited by KD (185). BHBA produced by KD may play a major role in these effects. In AD mice, BHB reduces microglia proliferation and activation (135, 186). In neuroinflammatory models, BHBA promotes microglia polarization toward M2, which exerts anti-inflammatory effects and prevents depressive-like behaviors (187). In spinal cord injury, BHB inhibits NLRP3 inflammasome and transfers the activation state of microglia from M1 to M2a phenotype, reducing neuroinflammation (188). However, some studies have also raised questions about the central role of ketone bodies. For instance, the medium-chain triglyceride KD is a commonly used variation (189). Medium-chain fatty acids may play a central role in the KD, which may regulate mitochondrial metabolism in astrocytes and have positive effects on the brain such as accelerated glycolysis, enhanced lactate shuttle, and accelerated ketone body production (190). Thus, KD may play a neuroprotective role by reducing the activation of glial cells, which is crucial in the pathogenesis of SUD. However, there is no direct evidence that KD attenuates glial cell activation in SUD, and further relevant studies are necessary.

## 7. Ketogenic diet modulate gut microbiota

Human microbes are mainly found in the gastrointestinal tract, including bacteria, fungi, viruses, etc. Gut microbes regulate neurological function and behavior and are associated with many neurological diseases and psychiatric disorders, such as multiple sclerosis, Parkinson’s disease, Alzheimer’s disease, Huntington’s disease, and amyotrophic lateral sclerosis (191), as well as depression, and anxiety (192).

Although not yet clear, there is growing evidence of a strong link between SUD and gut microbes (193–195). A study of the gut microbiota composition of patients with more than 10 years of alcohol overconsumption showed a higher relative abundance of bacteria from phylum *Proteobacteria* and higher levels of the genera *Sutterella*, *Holdemania*, and *Clostridium*, and a lower relative abundance of bacteria from genus *Faecalibacterium* compared to control patients with no or low alcohol intake history (196). Decreased fecal microbial alpha diversity in people with AUD who actively drink alcohol, characterized by a decrease in *Akkermansia* and an increase in *Bacteroides* (197). Chronic vapor alcohol exposure mice significant increases in genus *Alistipes* and significant reductions in genra *Clostridium* IV and XIVb, *Dorea*, and *Coprococcus* (198). However, another study showed that alcohol led to an opposite significant increase in gut microbial diversity in mice, and the abundance of phylum *Firmicutes* and class *Clostridiales* were elevated (167). Alcohol overconsumption leads to changes in the composition of the gut microbiota and appears to cause pro-inflammatory effects. Alcohol-induced neuroinflammation and gut inflammation in mice can be attenuated by reducing the bacterial load in the gut with antibiotics (138), suggesting that alcohol-induced inflammation may be related to gut bacteria. Methadone maintenance therapy leads to an imbalance of key bacterial communities, mainly *Akkermansia muciniphila*, required for the production of short-chain fatty acids, mucus degradation, and maintenance of barrier integrity. And *Bifidobacteriaceae* was significantly increased (199). Chronic morphine treatment significantly alters the gut microbial composition, decreases the *Bacteroidetes*/*Firmicutes* ratio, and induces amplification of gram-positive pathogenic bacteria (200). The heroin-dependent mouse model had reduced gut microbial alpha diversity with higher levels of *Bifidobacterium* and *Sutterella* and reduced levels of *Akkermansia* compared to controls (201). Cocaine causes alterations in the gut microbiota of mice and is associated with the upregulation of pro-inflammatory mediators, such as nuclear factor-kappa B and IL-1β (202). In addition, methamphetamine use disorders resulted in altered gut microbes, increased the relative abundance of pathogenic gut bacteria, and decreased the relative abundance of probiotic bacteria in mice, which was associated with systemic inflammation (203, 204). Based on the above studies, it appears that different types of SUD lead to some similar trends in gut microbes. Typically, *Sutterella* is significantly increased in alcohol and heroin use (196, 201), and *Bifidobacterium* is generally increased in opioid use (199, 201), and *Akkermansia* is generally decreased in alcohol and opioid use (197, 199, 201). Interestingly, an increased abundance of *Sutterella* may be associated with autism spectrum disorder (205). *Bifidobacterium* has been shown to induce the accumulation of gut pro-inflammatory Th17 cells (206). *Akkermansia* is associated with improved glucose homeostasis, modulation of the immune response, and protection of barrier function (207–209). Through mucus protein degradation, *Akkermansia* has beneficial effects on the gut barrier (210). Synbiotic treatment reduced the escalation and relapse to alcohol intake, possibly related to *Akkermansia* abundance restoration (211). However, it has also been shown that in a methamphetamine-induced CPP rat model, increased *Akkermansia* is associated with higher CPP scores (212). In addition, chronic alcohol feeding may lead to an increase in *Akkermansia muciniphila* in mice (213, 214). The opposite results seem to be related to the different types of substances of abuse and experimental subjects. Even so, it is clear that SUD causes widespread gut microbial disorders, resulting in a range of adverse consequences.

Gut microbial disorders resulting from SUD may also lead to behavior changes. Some studies have shown a close correlation between SUD-related behaviors and gut microbes. For example, dysbiosis of the gut microbiota during chronic alcohol exposure was strongly associated with alcohol-induced behaviors, and a decrease in the *Adlercreutzia* spp. was positively associated with alcohol preference and negatively associated with anxiety-like behavior (215). Methamphetamine-altered gut microbial composition is associated with depressive-like behavior (216). There is growing interest in using fecal microbiota to treat people with SUD. Through the treatment of fecal microbiota transplantation (FMT), the important role of gut microbes is revealed. In a double-blind randomized clinical trial, FMT from a donor enriched in *Lachnospiraceae* and *Ruminococcaceae* caused favorable gut microbial changes in patients with AUD-related cirrhosis and reduced alcohol craving and consumption (217). And a variety of addiction-related behaviors were shown to be associated with *Lachnospiraceae* and *Ruminococcaceae* (218). Gut microbes influence the development of morphine dependence, while FMT may reduce opioid withdrawal responses (219). In addition, transplantation by specific probiotics may also affect SUD-induced behavioral responses. For example, *Lactobacillus rhamnosus* probiotic can reduce cocaine-induced behavioral responses (220). Interestingly, transplantation of SUD-associated gut microbes into normal controls would result in behavioral changes similar to those associated with SUD. For instance, transplantation of gut microbiota from AUD patients into mice induced a decrease in BHBA metabolism that may be associated with social impairment and depression in AUD (221). Similarly, transplantation of gut microbes from alcohol-fed mice to healthy controls triggered anxiety behaviors similar to those induced by alcohol withdrawal (222). Thus, SUD alters gut microbes and induces related behaviors, suggesting that the gut microbiota may be an important regulator of SUD susceptibility.

Changes in gut microbiota associated with KD have been demonstrated to play a beneficial role in a variety of diseases. Importantly, the general decrease in *Akkermansia* in SUD may be reversed by KD. For example, in epilepsy, KD provides epilepsy protection by increasing the relative abundance of *Akkermansia* and *Parabacteroides* and by increasing the ratio of *Bacteroidetes*/*Firmicutes*, similarly, transplantation of KD-associated gut microbiota and treatment with *Akkermansia* and *Parabacteroides* also provide epilepsy protection in control mice (223, 224). In AD, KD also increased the relative abundance of beneficial gut microbiota (such as *Akkermansia muciniphila* and *Lactobacillus*) and decreased the relative abundance of pro-inflammatory microbiota (such as *Desulfovibrio* and *Turicibacter*) and improved metabolic conditions. They reduced the risk of Alzheimer’s disease and improved Alzheimer’s disease biomarkers in cerebrospinal fluid (225, 226). In colitis, 16 weeks of KD increases *Akkermansia* abundance, protects the gut barrier, and alleviates inflammation (227). Moreover, KD was able to reduce gut pro-inflammatory Th17 cell accumulation by inhibiting the growth of *Bifidobacterium* (206, 228). This may be part of the anti-inflammatory mechanism of KD.

However, some researchers have also raised concerns about the negative impact of KD on gut microbiota and health. In children with severe epilepsy, KD resulted in a significant decrease in the relative abundance of *Bifidobacteria* as well as *E. rectale* and *Dialister*, and an increase in the relative abundance of *E. coli*, indicating a decrease in the abundance of health-promoting fiber-consuming bacteria (229). Similarly, KD pre-treatment for 1 month resulted in an increase in gut pathogenic bacteria and a decrease in beneficial bacteria in inflammatory bowel disease mice, which increased gut and systemic inflammation, disrupted the gut barrier, and exacerbated colitis (230). These different outcomes resulting from KD may be attributed to different populations, diseases, and treatment protocols. The exact reasons need to be further explored, given that different gut microbial alterations have been observed in different diseases. However, certainly, KD may partially regulate gut microbial dysbiosis caused by SUD, such as *Akkermansia* dysbiosis.

## 8. Limitations

Various effects of KD may be beneficial for recovery from SUD. However, there are few reports of KD being used for the treatment of SUD besides AUD. Also, some researchers have shown concerns during the application of KD in neurological diseases, such as decreased appetite, increased risk of malnutrition, and the occurrence of some adverse effects (231). Common KD adverse effects include metabolic abnormalities, gastrointestinal symptoms, kidney stones, and slow growth in children (232). However, most of these adverse effects were evaluated in children. Even so, KD should be applied with caution in people with SUD, as this particular population often suffers from multisystem disorders such as the increased risk of malnutrition (36) and gastrointestinal symptoms (233).

## 9. Conclusion

The successful exploration of the KD in other neurological disorders suggests a positive role in SUD that still cannot be ignored. It may play a positive role in the improvement of various disorders caused by SUD, especially the reduction of sugar intake, alteration of metabolic processes, improvement of neural circuits associated with substance addiction, neuroprotective effects, improvement of glial cell activation, and modulation of gut microbiota (Table 2). In conclusion, there are potential therapeutic implications of the KD in SUD, but many problems remain. Further preclinical studies and clinical randomized controlled trials are needed to investigate and improve strategies for KD, such as optimization of the timing of interventions and nutrient composition, to assess the suitability, effectiveness, and safety of KD in the treatment of SUD.

**Table 2:** Pathology of SUD and potential targeted benefits of KD.

Pathology of SUD	Substance	Potential targeted benefits of KD
Sugar consumption↑	Opiate (37); Heroin (38); Methadone (39); Cocaine (41); Alcohol (42, 43)	Carbohydrates intake↓ (May improve the neural circuits that encode reward)
Neurotransmitter imbalances (Glutamate/GABAergic system dysfunction)	Cocaine (83–85); Heroin (86); Nicotine (87); Alcohol (88–90); Methamphetamine (234)	Glutamate↓, GABA↑ (95–98, 101, 102)
Brain glucose metabolism↓; Acetate uptake↑	Alcohol (28, 30–32, 103); Opiate (107); Methadone (108); Cocaine (109); Nicotine (110); Methamphetamine (111–113)	Ketone metabolism↑ (15, 16)
Neuronal damage	Heroin (119, 120); Methamphetamine (121, 122, 124); Cocaine (123)	Neuroprotection (mitochondrial function↑; ROS↓; oxidative stress↓) (125, 126, 129–136)
Inflammation↑	Alcohol (138); Morphine (139); Cocaine (140); Methamphetamine (147); Heroin (235); Methadone (236)	Inflammation↓ (148–151, 153, 154)
Glial cell dysfunction	Alcohol (167, 168); Morphine (169, 180, 181); Cocaine (170, 171); Methamphetamine (142); Heroin (237)	Glia cell inflammatory activation↓ (135, 182–188)
Gut microbiota disorders	Alcohol (138, 167, 196–198, 211, 213–215, 217, 221, 222); Methadone (199); Morphine (200, 219); Heroin (201); Cocaine (202); Methamphetamine (203, 204, 212, 216)	Modulate gut microbiota (206, 223–228)

↑: increased; ↓: decreased; SUD, substance use disorders; KD, ketogenic diet; GABA, γ-aminobutyric acid; ROS, reactive oxygen species.

## Author contributions

DK, J-xS, and J-qY: conceptualization, original draft preparation, review, and editing. Y-sL, KB, Z-yZ, and K-hW: conceptualization, review, and editing. H-yL, MZ, and YX: review and editing. All authors contributed to the article and approved the submitted version.

## Funding

This work was partly supported by the Yunnan Technological Innovation Centre of Drug Addiction Medicine (202305AK340001); the Biomedical Science Resource Database of Kunming Medical University (Drug Abuse Population Cohort; Digitalization, Development and Application of Biotic Resource) (No. 202002AA100007); the National Natural Science Foundation of China (No. 81860100); the high-level health technology talent reserve in Yunnan Province (No. H-2018062); the Yunnan Province “high-level talent training support program” training program (Nos. YNWR-QNBJ-2019-243 and RLMY20200019); the Reserve Talent Project for Young and Middle-aged Academic and Technical Leaders of Yunnan Province Science and Technology Department (No. 202005AC160057); the Huang Chang-ming Expert Workstation of Yunnan Province (No. 202005AF150090); and the Yunnan Province and the Education Department of Yunnan Province (No. 2019J1226).

## Conflict of interest

The authors declare that the research was conducted in the absence of any commercial or financial relationships that could be construed as a potential conflict of interest.

## Publisher’s note

All claims expressed in this article are solely those of the authors and do not necessarily represent those of their affiliated organizations, or those of the publisher, the editors and the reviewers. Any product that may be evaluated in this article, or claim that may be made by its manufacturer, is not guaranteed or endorsed by the publisher.

## Abbreviations

AUD: alcohol use disorders
BHB: β-hydroxybutyric
CPP: conditioned place preference
DA: dopamine
DUD: drug use disorders
GABA: γ-aminobutyric acid
IL: interleukin
KD: ketogenic diet
MOR: mu-opioid receptor
NAc: nucleus accumbens
NLRP3: nucleotide-binding domain-like receptor protein 3
ROS: reactive oxygen species
SUD: substance use disorders
TNF: tumor necrosis factor
